# Palms of the past: can morphometric phytolith analysis inform deep time evolution and palaeoecology of Arecaceae?

**DOI:** 10.1093/aob/mcae068

**Published:** 2024-04-30

**Authors:** W H Brightly, C Crifò, T J Gallaher, R Hermans, S Lavin, A J Lowe, C A Smythies, E Stiles, P Wilson Deibel, C A E Strömberg

**Affiliations:** Department of Biology, University of Washington, Seattle, WA 98195, USA; Burke Museum of Natural History and Culture, University of Washington, Seattle, WA 98195, USA; Department of Plant and Animal Sciences, University of Sheffield, SheffieldS10 2TN, UK; Conservatoire d’espaces naturels de Provence-Alpes Côte d’ Azur – Maison de la Crau, 13310 Saint-Martin de Crau, France; Bernice Pauahi Bishop Museum, Honolulu, HI 96817, USA; Archaeology, Environmental Changes and Geo-Chemistry (AMGC), Vrije Universiteit Brussel, Brussels 1050, Belgium; Department of Biology, University of Washington, Seattle, WA 98195, USA; Burke Museum of Natural History and Culture, University of Washington, Seattle, WA 98195, USA; Department of Ecology and Evolution, Stony Brook University, Stony Brook, NY 11794, USA; Department of Biology, University of Washington, Seattle, WA 98195, USA; Burke Museum of Natural History and Culture, University of Washington, Seattle, WA 98195, USA; Department of Biology, University of Washington, Seattle, WA 98195, USA; Burke Museum of Natural History and Culture, University of Washington, Seattle, WA 98195, USA; Department of Biology, University of Washington, Seattle, WA 98195, USA; Burke Museum of Natural History and Culture, University of Washington, Seattle, WA 98195, USA; Burke Museum of Natural History and Culture, University of Washington, Seattle, WA 98195, USA; Department of Biology, University of Washington, Seattle, WA 98195, USA; Burke Museum of Natural History and Culture, University of Washington, Seattle, WA 98195, USA

**Keywords:** Arecaceae, palm, phytolith, palaeobotany, palaeoecology, morphometric, phylogenetic comparative methods

## Abstract

**Background and aims:**

Palm fossils are often used as evidence for warm and wet palaeoenvironments, reflecting the affinities of most modern palms. However, several extant palm lineages tolerate cool and/or arid climates, making a clear understanding of the taxonomic composition of ancient palm communities important for reliable palaeoenvironmental inference. However, taxonomically identifiable palm fossils are rare and often confined to specific facies. Although the resolution of taxonomic information they provide remains unclear, phytoliths (microscopic silica bodies) provide a possible solution because of their high preservation potential under conditions where other plant fossils are scarce. We thus evaluate the taxonomic and palaeoenvironmental utility of palm phytoliths.

**Methods:**

We quantified phytolith morphology of 97 modern palm and other monocot species. Using this dataset, we tested the ability of five common discriminant methods to identify nine major palm clades. We then compiled a dataset of species’ climate preferences and tested if they were correlated with phytolith morphology using a phylogenetic comparative approach. Finally, we reconstructed palm communities and palaeoenvironmental conditions at six fossil sites.

**Key results:**

Best-performing models correctly identified phytoliths to their clade of origin only 59 % of the time. Although palms were generally distinguished from non-palms, few palm clades were highly distinct, and phytolith morphology was weakly correlated with species’ environmental preferences. Reconstructions at all fossil sites suggested that palm communities were dominated by Trachycarpeae and Areceae, with warm, equable climates and high, potentially seasonal rainfall. However, fossil site reconstructions had high uncertainty and often conflicted with other climate proxies.

**Conclusions:**

While phytolith morphology provides some distinction among palm clades, caution is warranted. Unlike prior spatially restricted studies, our geographically and phylogenetically broad study indicates phytolith morphology may not reliably differentiate most palm taxa in deep time. Nevertheless, it reveals distinct clades, including some likely to be palaeoenvironmentally informative.

## INTRODUCTION

The palm family (Arecaceae) has one of the richest and best studied fossil records of any group of flowering plants with a wealth of fossilized pollen, leaves, fruits, seeds, and stems from the Early Cretaceous onward (Harley and Baker, 2001; Kvaček and Herman, 2004; Harley, 2006; Matsunaga *et al.*, 2019). By the Palaeogene, all five extant palm subfamilies are known from the fossil record, with the family having achieved a worldwide distribution (e.g. Taylor, 1990; Mustoe and Gannaway, 1995; Harley, 2006; Iles *et al.*, 2015; Greenwood and West, 2017; Grímsson *et al.*, 2019; Greenwood and Conran, 2020; Huang *et al*., 2020). Today, over 90 % of palm species diversity is concentrated in tropical rainforests and wetlands (Couvreur and Baker, 2013), and most palm taxa cannot reproduce in climates with prolonged periods of freezing temperatures (Archibald *et al.*, 2014; Reichgelt, *et al.*, 2018). Under the assumption that the ecology of ancient palms closely resembled that of most living taxa, the presence of palm fossils has often been interpreted to indicate warm – and wet – climates in Earth’s past (Wolfe, 1977; Wing and Greenwood, 1993; Greenwood and Wing, 1995; Archibald *et al.*, 2014; Reichgelt *et al.*, 2018; Su *et al.*, 2019).

Several recent observations challenge the status of palms as unequivocal markers of warm and wet conditions, highlighting the need for a more nuanced approach to ecological inferences using Arecaceae fossils. First, although their diversity is highest in warm and wet regions, the geographic distribution of modern palms demonstrates that the temperature tolerance of palms is variable (Reichgelt *et al.*, 2018). Although generally less speciose, certain extant palm lineages consistently occupy cooler and/or drier regions. Species within the tribe Trachycarpeae, for example, are among the most tolerant of cooler temperatures (Couvreur *et al.*, 2011; Reichgelt *et al.*, 2018). Second, integrated climatic–phylogenetic analyses (Couvreur *et al.*, 2011; Baker and Couvreur, 2013; Greenwood and West, 2017; Cano *et al.*, 2018; Cássia-Silva *et al.*, 2019) and the abundance or dominance of palms in a range of palaeoenvironments (e.g. Mustoe and Gannaway, 1995; Breedlovestrout *et al.*, 2013; Dunn *et al.*, 2015) suggest ancient palms may have played a more important ecological role in a broader range of environments than today. This potential discrepancy between modern and ancient Arecaceae means that fossil palms cannot automatically be interpreted as indicating tropical rainforest conditions. Instead, to use palm fossils for constraining environmental conditions based on nearest living relative approaches, it is crucial to determine which palm lineage is represented.

The resolution of taxonomic information that can be obtained from palm fossils varies with the organ preserved (e.g. leaves, fruit, stem) and quality of preservation. Fossil palm pollen is likely the earliest record of palms and is widespread by the Maastrichtian (Harley and Baker, 2001; Harley, 2006; Martinez *et al.*, 2016). However, identification of fossil pollen below the family level is often difficult, as most palms have simple tectate and monosulcate pollen, and very few genera have diagnostic pollen morphology (e.g. *Mauritia*, *Arenga*, *Eugeissona*, *Nypa*; Gee, 2001; Harley, 2006). Similarly, palm stems, reported as early as the Turonian of France (Berry, 1911; Kvaček and Herman, 2004), are typically not diagnostic below the family level (Harley, 2006; although see Thomas and De Franceschi, 2012). Impressions and compressions of palm leaves (fronds) are abundant in the fossil record and can facilitate reliable assignments to subfamily and tribe, but only in the rare cases when high-order veins and epidermal anatomy are preserved (Daghlian, 1981; Kvaček and Herman, 2004). Reproductive organs (e.g. fruits and seeds) often allow assignment to tribe or lower taxonomic levels, but are comparatively rare as fossils (e.g. Manchester *et al.*, 2010; Matsunaga *et al.*, 2019; Matsunaga and Smith, 2021). Notably, the preservation of taxonomically diagnostic traits in leaves and reproductive structures requires specific conditions, such as anoxia and rapid sedimentation, which is best achieved in lake and swamp environments, severely limiting the contexts from which taxonomically identifiable palm fossils can be studied.

In recent years, phytoliths (microscopic silica bodies) have emerged as an additional source of information about fossil palms (e.g. Mazzoni, 1979; Pinilla and Bustillo, 1997; Strömberg, 2004; Zucol *et al.*, 2010). Phytoliths form when hydrated silica is precipitated in the tissues of many land plants and can remain in the soil or sediment after the plant litter decays (Strömberg *et al.*, 2018). Palms produce phytoliths in specialized silica-secreting cells (stegmata) which are found in longitudinal files of cells adjacent to vascular or non-vascular fibres (Tomlinson, 1961). Stegmata-derived phytoliths have easily recognizable shapes, and have therefore long been regarded as a dependable indicator of the past presence of palms (e.g. Mazzoni, 1979; Alexandre *et al.*, 1997; Piperno, 2006; Albert *et al.*, 2009; Morcote-Ríos *et al.*, 2016). Because of their relatively inert and robust composition, phytoliths also substantially broaden the types of facies that routinely yield palm and other plant fossils to include well oxidized palaeosols and aeolian or volcaniclastic sediments and sedimentary rocks, providing a more complete sampling of past environments (Mösle *et al.*, 1998; Strömberg, 2002; Strömberg *et al.*, 2018). These characteristics – the diagnostic morphologies and broad fossil occurrence – give phytoliths considerable promise as a tool for studying palm palaeoecology. However, the taxonomic resolution of palm phytoliths, particularly in deep time contexts, is poorly understood.

**Fig. 1.**
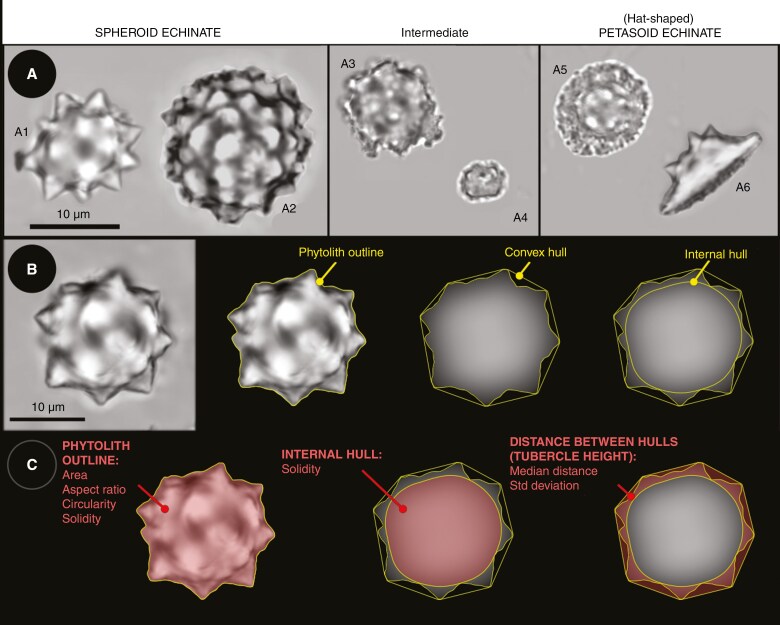
Example phytoliths and general procedure for extracting morphometric features from phytoliths. (A) Examples of each of the general morphotypes described in the text. Note that a6 shows an oblique view of a hat-shaped (Petasoid echinate; see Results). phytolith to illustrate this type’s 3-D morphology, but measurements were only taken from phytoliths photographed from a dorsal view (as in A5). Taxa represented are (A1) *Plectocomia elongata*, (A2) *Rhapis excelsa*, (A3) *Orania palindan*, (A4) *Bactris gasipaes*, (A5) *Nypa fruticans* and (A6) *Iriartella stenocarpa*. (B) Phytolith of *Calamus rhabdocladus* through the various stages of processing described in the text to extract morphological features. Using the same phytolith, panel (C) illustrates the seven quantitative morphological features used in discriminant analyses.

Stegmata-derived palm phytoliths are typically divided into two main morphotypes (Fig. 1A), ‘echinate spheres’ (‘Spheroid echinate’ in Neumann *et al.*, 2019) and ‘hats’ (e.g. Tomlinson, 1961, Piperno, 1985, 1988; Alexandre *et al.*, 1997). More rarely, phytoliths appearing intermediate between these two morphotypes are also produced (pers. obs. CAE Strömberg; Fig. 1A). The Spheroid echinate type is commonly encountered in Cenozoic strata and has typically been viewed as an indicator of closed habitats or warm, and often wet, climates (e.g. Strömberg, 2004; Punyasena, 2008; Strömberg *et al.*, 2013). For example, in the Eocene of North America and Turkey, the occurrence of Spheroid echinate phytoliths together with other phytoliths diagnostic of woody dicotyledons was interpreted as pointing to (sub)tropical forest vegetation (Strömberg, 2005; Strömberg *et al.*, 2007; Miller *et al.*, 2012). In contrast, at Holocene-aged sites in regions where extant palms tend to be less tolerant of shade (e.g. tropical Africa, New Guinea), palm phytoliths have also been used to infer open or disturbed habitats (e.g. Boyd *et al.*, 1998; Runge, 1999). Hat-shaped palm phytoliths, although rarer in fossil assemblages, have often served to signal the presence of mangrove palm (*Nypa fruticans*) in sub-Recent sediments (e.g. Das *et al.*, 2014; Chabot *et al.*, 2018), although hat-shaped phytoliths are known to occur in other palms as well (Tomlinson, 1969). However, the distribution of Spheroid echinate phytoliths and hats among different palm subclades has not been well characterized in a phylogenetic context. In addition, morphologically similar phytoliths occur in several other monocot families (e.g. Prychid *et al.*, 2003; Benvenuto *et al.*, 2015). For these reasons, the detailed taxonomic affinities of fossil palm phytoliths remain uncertain and render ecological inferences difficult. This problem is particularly severe for pre-Holocene palms, which cannot necessarily be assumed to be closely related to modern taxa growing locally.

To address this problem, we evaluate the utility of stegmata-derived phytoliths to distinguish lower taxonomic groups within Arecaceae, and the degree to which they can be distinguished from non-palms producing similar phytolith morphologies. To quantify phytolith morphology, we adopt a morphometric approach. Similar approaches have already been shown to be effective in discriminating among phytoliths produced by closely related species mainly in the Poaceae (e.g. Pearsall *et al.*, 1995; Zhao *et al.*, 1998; Ball *et al.*, 1999; Berlin *et al.*, 2003; Portillo *et al.*, 2006; Zhang *et al.*, 2011; Out *et al.*, 2014; Out and Madella, 2015; Gallaher *et al.*, 2020; Hošková *et al.*, 2021) but also within other taxa (e.g. Ball *et al.*, 2006; Vrydaghs *et al.*, 2009). Several studies have also suggested that, within Arecaceae, differentiation of palm phytoliths at lower taxonomic level is possible using morphometric analysis (Albert *et al.*, 2009; Delhon and Orliac, 2010; Fenwick *et al.*, 2011; Bowdery, 2014; Benvenuto *et al.*, 2015; Morcote-Ríos *et al.*, 2016; Huisman *et al.*, 2018; Bokor *et al.*, 2019; Witteveen *et al.*, 2022). In addition, Benvenuto *et al.* (2015) showed that size can be used to discriminate Spheroid echinate phytoliths occurring in Arecaceae from similar morphotypes occurring in Bromeliaceae. Nevertheless, a general consensus on the diagnostic characters of phytoliths within Arecaceae is lacking, mainly because (1) previous studies are based on differing morphological variables (sometimes combined with qualitative characters); and (2) overall, they cover only a small fraction of the family’s diversity by focusing on a limited number of taxa (e.g. Albert *et al.*, 2009, Delhon and Orliac, 2010, Fenwick *et al.*, 2011, Bowdery, 2014, Benvenuto *et al.*, 2015; Morcote-Ríos *et al.*, 2016; Huisman *et al.*, 2018; Witteveen *et al.*, 2022). Therefore, they are less useful for deep time ecological inferences, which cannot rely as heavily on comparison with modern palm communities of the same region. Here we expand the taxonomic and geographic scope of previous morphometric work by applying a standard set of shape measures to a global sample of species to test for distinctiveness of phytolith morphology within the Arecaceae. We further test the approach using fossil phytoliths preserved at six Eocene–Miocene-aged sites spanning three continents.

## MATERIALS AND METHODS

### Sampling protocol and preparation

Within Arecaceae, we sampled 87 species representing 87 of 181 genera, 27 of 28 tribes, and all five subfamilies (Baker and Dransfield, 2016). In addition, we sampled ten species from four additional families (Bromeliaceae, Marantaceae, Orchidaceae and Zingiberaceae) chosen on the basis of similarity of phytolith morphology to the palms (e.g. Tomlinson, 1969; Piperno, 1985; Zhou, 1995; Chen and Smith, 2013). Leaf samples for phytolith extraction were collected from the Fairchild Botanical Garden (FTG; 60 samples), the herbarium of Missouri Botanical Garden (MO; 17 samples), the New York Botanical Garden (NYBG; 4 samples), the Rancho Santa Ana Botanical Garden (RSA; 1 sample) and the University of California Jepson Herbarium (UCJH; 1 sample) (see Supplementary Data Table S1 for a detailed list of samples).

To test our classification method on palaeontological samples, we used six previously published fossil phytolith assemblages from Argentina, Turkey and the USA: namely, samples from the Middle Eocene Gran Barranca Member (here ARG1), the Late Eocene Vera Member (ARG2) and the Early Miocene UPA Member (ARG3), all of the Sarmiento Formation, Gran Barranca, Patagonia Argentina (Strömberg *et al.*, 2013; Dunn *et al.*, 2015); unnamed Middle Eocene strata (Ankara-Çubuk-Susuzköy; TUR1) and the Middle Miocene Ardıç Formation (Ardiç-Mordogan-Izmir; TUR2) of Turkey (Strömberg *et al.*, 2007); the Late Eocene Peanut Peak Member of the Chadron Formation, Nebraska, USA (USA1) (see Supplementary Data Table S1 for details). All assemblages contain abundant, well-preserved Spheroid echinate phytoliths, while TUR2 also has low abundances of hat-shaped palm phytoliths. In all cases, the presence of Spheroid echinate phytoliths has been used to infer warm–wet, more or less closed forest habitats (Strömberg, 2005; Strömberg *et al.*, 2007, 2013), although palms were later inferred to have been dry-adapted in the samples from Argentina based on independent evidence (a phytolith shape-based openness proxy, isotope-based regional climate data, and sedimentology; Bellosi and González, 2010; Dunn *et al.*, 2015; Kohn *et al.*, 2015). The hat-shaped phytoliths from TUR2 were not originally distinguished from Spheroid echinate forms, nor were their potentially different ecological implications discussed (Strömberg *et al.*, 2007).

For extraction of phytoliths from modern tissues, we used dry leaves (0.025 g per sample) and followed standard extraction procedures (Piperno, 1988; Strömberg, 2003). Phytoliths were extracted from sedimentary rock samples following methods described in Strömberg *et al.* (2007). With all samples, the extracted silica was mounted on slides using a fixed mounting medium (Cargille MeltMount^®^) for microscope observation and imaging. Vials and slides were deposited in the University of Washington Burke Museum’s Paleobotany Reference and Paleobotany Fossil collections (Supplementary Data Table S1).

### Imaging and measurement

Phytoliths were imaged with a Leica TCS SP5 (Wetzlar, Germany) or Nikon A1 (Tokyo, Japan) confocal microscope, with a Plan Apo CS ×63 or Plan Apo λ ×60 oil immersion lens, respectively. Phytolith images were taken in z-stacks with step size 0.13 or 0.1 μm, with at least 30 phytoliths imaged for each extant species or fossil assemblage sampled. Full details of microscopy are provided in the Supplementary Data Methods S1. Images of individual phytoliths were analysed and measured in ImageJ (Schneider *et al.*, 2012) using a custom macro. In brief, this extracts standard shape and size descriptors from a user-defined phytolith outline and ‘internal hull’ (extending to the deepest sinus between two tubercles), and records typical tubercle size and variation from the distance between the internal hull and the convex hull of the phytolith outline (Fig. 1B, Supplementary Data Methods S2).

### Taxonomic groups and primary discriminant analyses

**Fig. 2.**
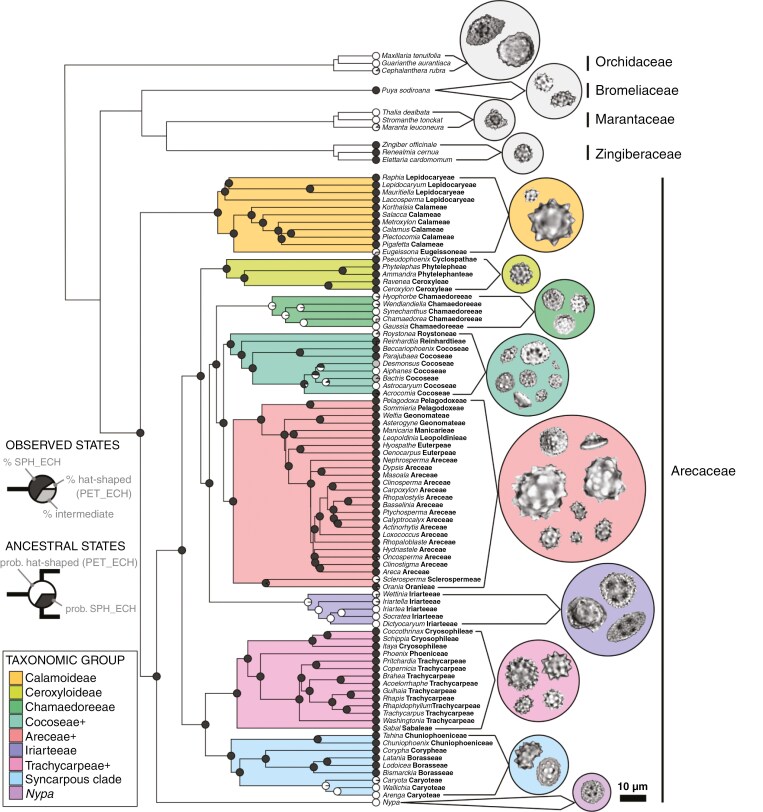
Taxonomic groups used in discriminant analyses (coloured clades), their phylogenetic relationships (following Faurby *et al.*, 2016) and examples of the phytoliths produced by each. Tribal affinities (bold text) within Areceae are given for each genus. The observed proportion of each phytolith morphotype (sph_ech = Spheroid echinate, pet_ech = Petasoid echinate) is indicated for sampled taxa (pie charts at tips), and probability of producing each morphotype based upon ancestral state estimates is given at interior nodes (within the Arecaceae only).

For discriminant analysis, taxa were initially grouped by subfamily, with additional division to minimize class imbalance, an issue that often results in classifiers biased towards larger groups (Japkowicz and Shaju, 2002; Johnson and Khoshgoftaar, 2019). The species richness of palm subfamilies varies by multiple orders of magnitude. The large subfamilies Arecoideae and Coryphoideae were thus divided into smaller groups largely following tribal boundaries, while enforcing minimum group size and monophyly. Outgroup taxa were all treated at the family level. This resulted in a scheme of 13 taxonomic groups (Fig. 2). Additional analyses based upon strict subfamily and tribe boundaries were also conducted.

Discriminant analyses incorporated eight predictors selected to capture size and shape while minimizing collinearity of predictors. These were (1) phytolith morphotype (Spheroid echinate, hat-shaped or intermediate), (2) phytolith area, (3) phytolith circularity (4π × [area/perimeter^2^]), (4) phytolith aspect ratio (major/minor axis), (5) phytolith solidity (area/convex hull area), (6) solidity of the internal hull, (7) median tubercle height, and (8) standard deviation of tubercle height (Fig. 1C).

Previous work has shown that discriminant methods vary in effectiveness depending on the nature of the classification problem (Holden *et al*., 2011). We thus tested five common discriminant methods to assess how diagnostic phytolith morphology was of taxonomic group within the Arecaceae. Methods were chosen on the basis of their popularity in the literature and incorporation of a diverse set of underlying statistical approaches (e.g. Shihong *et al.*, 2003; Holden *et al.*, 2011; Qi, 2012; Gallaher *et al.*, 2020; Weaver *et al.*, 2022). The first method, linear discriminant analysis (LDA), is one of the most widely used discriminant methods. It operates by finding the linear combination of predictors that maximizes distance between groups (Holden *et al.*, 2011). Mixture discriminant analysis (MDA) modifies this approach by treating each group as comprising several unobserved subgroups, which allows the model greater flexibility in capturing the distribution of values within each group (Hastie and Tibshirani, 1996; Holden *et al.*, 2011). Logistic regression (LR) fits a logit function representing the probability of belonging to a group given some predictor values (Holden *et al.*, 2011). Although classic LR predicts a binary response, multinomial LR (mLR) extends this to cases with more than two groups. Random forest (RF) is a decision-tree-based algorithm, which increases performance through bootstrapping, basing classifications on a large number of decision trees constructed from random samples of the predictors (Breiman, 2001; Holden *et al.*, 2011). The final method, support vector machines (SVM), uses a linear classifier to separate groups in space, allowing for the maximum margin between them, and incorporating a kernel function to project data into higher dimensions when they cannot be easily separated in lower-dimensional space (Noble, 2006).

Prior to model fitting, the dataset was split into two partitions, with the larger (70 % of data, 1781 phytoliths) used for model training and the smaller (30 % of data, 753 phytoliths) retained to evaluate model performance. Training included ten repeats of 10-fold cross-validation, with the best model chosen by maximizing Cohen’s *κ*. Cohen’s *κ* is a performance metric that takes into account correct classifications occurring by chance and is generally considered more robust than overall accuracy when sample sizes differ between groups (Cohen, 1960; McHugh, 2012; although see Delgado and Tibau, 2019). Resulting models were used to make predictions from phytoliths reserved in the test partition, and these predictions were then compared with their known group identity. The five methods were compared on the basis of overall accuracy and *κ*, as well as group-specific precision (i.e. proportion of correct guesses) and recall (i.e. proportion of phytoliths correctly identified). All analyses were completed in the R software environment (v. 4.1.3; R Core Team, 2022) using the *caret* package (v. 6.0-92; Kuhn, 2022) and as a wrapper for following packages: *mda* (Leisch *et al.*, 2020); *randomForest* (Liaw and Wiener, 2002); and *kernlab* (Karatzoglou *et al.*, 2004). Following model fitting, we used *caret*’s varImp function to evaluate the relative importance of morphological predictors on the basis of the accuracy reduction if excluded from the model.

### Additional discriminant analyses

Because not all discriminant methods natively support categorical predictors (LDA, MDA, SVM), morphotype data could not be included in some analyses. Thus, we also tested separate models for each morphotype (Spheroid echinate, hat-shaped), and evaluated whether these models treated as an ensemble (i.e. using two models in conjunction to make predictions) improved overall performance relative to our primary analyses. Because intermediate phytolith morphotypes were not numerous enough to train separate models, intermediates were grouped depending on the other morphotype produced by the species in question (none of the sampled taxa were observed to produce both Spheroid echinate and hat-shaped phytoliths).

We also reran analyses with upsampling of small groups and downsampling of large groups during model cross-validation to test whether it helped alleviate the effects of class imbalance. To conduct these analyses, we used default sampling procedures in the *caret* package, which draw random samples from each group to match the size of the smallest (downsampling) and largest (upsampling) groups, respectively (Kuhn, 2022).

### Ecological data and comparative analyses

To facilitate palaeoenvironmental reconstructions at fossil sites, we evaluated the range of environmental conditions each of our taxonomic groups occupied (see Fossil predictions and nearest living relative analyses section, below). We downloaded all available georeferenced records for the palm family from the Global Biodiversity Information Facility (GBIF, 2022), and used GBIF flags and the R package *CoordinateCleaner* (v. 2.0-11; Zizka *et al.*, 2019) to discard those with low-quality taxonomic or geospatial data (Supplementary Data Methods S3). We also discarded occurrences of all genera and cultivated species falling outside their known native ranges (Reichgelt *et al.*, 2018; Plants of the World Online), and kept only unique species-site occurrences (i.e. to prevent well populated or studied sites from disproportionately influencing results; Reichgelt *et al.*, 2018). All species with five or fewer records were discarded.

Using vetted occurrence records, we extracted mean annual temperature (MAT), mean annual precipitation (MAP), minimum temperature of the coldest month (CMT) and precipitation of the driest quarter (DQP) from WorldClim 2 climate surfaces (Fick and Hijmans, 2017). For each species, we also recorded the proportion of occurrences found in evergreen forests using v. 2.07 of the European Space Agency (ESA) Climate Change Initiative Land Cover (CCI-LC) maps (300-m resolution, 1992–2015; ESA, 2021). For the latter, we did not include anthropogenic habitats (e.g. those classed as urban or agricultural) in calculations, and used habitat type observed for the longest period when there was variation over time. Occurrence records and environmental data are provided in Supplementary Data Table S2.

To investigate the evolution of habitat preference within the family, we conducted ancestral state estimation of all climate and habitat variables. For these analyses, we used a maximum clade credibility tree of the palm family, constructed using TreeAnnotator (Drummond *et al.*, 2012) from the posterior distribution of trees from Faurby’s conservative constraints analysis (Faurby *et al.*, 2016). Reconstructions of climate variables were conducted using the R package *Rphylopars* (v. 0.3.2, Goolsby *et al.*, 2021), which enables multiple traits and observations per trait to be passed to each tip of the phylogeny, and variation within species and correlation between traits to be incorporated into reconstructions. All species for which we obtained climate data were included in this analysis. Ancestral state estimates were conducted under a Brownian motion, Ornstein–Uhlenbeck, early burst, and a lambda model of trait evolution. The Akaike information criterion (Sakamoto *et al.*, 1986) suggested lambda models provided the best fit, and these were used in subsequent analyses. Ancestral state estimates of evergreen forest affinity (EFA) were conducted separately using the fastANC function in the *phytools* package (v. 2.0-3; Revell, 2012) since this trait contains only a single measure per species.

To test whether morphometric features were correlated with the environmental preferences of species, we constructed phylogenetically informed Bayesian mixed models using the R package *brms* (v. 2.15.0; Bürkner, 2017). These analyses allowed us to explore the possibility of reconstructing the palaeoenvironment from phytolith morphology directly (e.g. Dunn *et al.*, 2015), in addition to the alternative of using a nearest living relative approach (see next section). Models included MAT, CMT, MAP, DQP and EFA as fixed effects predicting each morphometric feature (precipitation variables were square-root-transformed). Response distributions were modelled as log-normal for phytolith area, aspect ratio and standard deviation of tubercle height, Gaussian for phytolith circularity, solidity and median tubercle height, and skew normal for the solidity of the internal hull. Phylogenetic relationships were accounted for by including species as a grouping factor with a phylogenetic covariance matrix (*ape* v. 5.4; Paradis and Schliep, 2019). Four chains of 3000 generations were run, with every other generation sampled and the first 600 discarded as burn-in. Weakly informative priors were assigned to all model parameters (Supplementary Data Fig. S1).

### Fossil predictions and nearest living relative analyses

For fossil phytoliths included in our dataset, we predicted group identity using the best-performing (see Results section) discriminant model from our modern dataset. In addition to these raw model predictions, we also conducted biogeographically informed predictions that disallowed predictions of taxonomic groups at sites outside their likely biogeographic ranges. Specifically, this disallowed the syncarpous clade from all North and South American sites, *Nypa* from post-Eocene sites in the Americas, and the Ceroxyloideae, Chamaedoreeae and Iriarteeae from both sites in Turkey (Gee, 2001; Baker and Couvreur, 2013).

To explore the palaeoenvironmental implications of our phytolith classifications, model predictions of taxonomic groups for fossil Spheroid echinate, intermediate and hat-shaped phytoliths were used to estimate site conditions using a coexistence approach. This method reconstructs climate based upon the range of conditions under which extant members of the groups identified at the fossil site can coexist (Mosbrugger and Utescher, 1997; Utescher *et al.*, 2014). Despite its limitations (e.g. Grimm and Potts, 2016), the coexistence approach is widely used (e.g. Zaborac-Reed and Leopold, 2016; Martínez *et al.*, 2020, 2021) and has the advantage of being straightforward to apply to any fossil assemblage with taxonomic identification. For each site, we recorded the range of conditions each unique taxonomic group occupied in our ecological dataset. The range of possible site conditions was then defined by the climate space in which all observed groups overlapped. To evaluate uncertainty imposed by assemblage size and model uncertainty, we compiled 100 bootstrap replicates of each phytolith assemblage, with fossil phytolith identity sampled five times per replicate with probability determined by the discriminant model output (i.e. a total of 500 assemblages per site). Coexistence analyses were conducted on the resulting set of bootstrapped assemblages.

We adopted a different approach for our habitat variable, EFA. Evergreen forest affinity values are proportions, and therefore do not reflect measurable site conditions in the same way as other environmental variables (i.e. the site was either an evergreen forest or some other habitat). For these data, we computed the probability of each group being present at a given fossil site (i.e. 1 minus the cumulative probability that none of the phytoliths belong to a given group). We then drew 100 samples per site from our ecological dataset, conditioned upon these presence/absence probabilities. The resulting distribution represents the range of EFA observed within groups likely to have been present at the sites, with greater emphasis placed on those groups for which there is stronger evidence of their presence. If the distribution of values compiled for a site is shifted towards higher evergreen forest affinities, this suggests the majority of extant species among groups likely found at the site primarily occur within evergreen forest habitats. Site data can thus be thought of as proportional to the probability that the site itself was an evergreen forest.

For ecological analyses, in order to maximize the available data, we included all taxa within each taxonomic group that met our vetting criteria regardless of whether they featured in our phytolith dataset. Environmental data were generally treated at the species level. However, because *Nypa fruticans* is the only extant member of the subfamily Nypoideae, environmental sampling within this group was intraspecific to allow some variation to be incorporated into reconstructions. Finally, because our dataset was compiled to evaluate patterns within the palm family, we discarded all fossil phytoliths that were assigned a high probability of belonging to one of the outgroup families (defined as greater than the model prior of a phytolith belonging to that group).

## RESULTS

### Phytolith dataset

In total we measured 2534 phytoliths in our modern dataset. Of these, ~70 % (1788) were Spheroid echinate, *~*25 % (626) were hat-shaped and the remainder (~5 %, 120) were intermediate between these two morphologies. The vast majority (86 %) of the latter were ‘hat-like’, being produced by taxa otherwise possessing hat-shaped phytoliths, or whose closest relatives produced hat-shaped phytoliths (Fig. 2). Ancestral state estimates show that production of hat-shaped phytoliths evolved several times within the Areceae, but was limited to the following seven lineages: Bactridinae (excluding *Acrocomia*), Caryoteae, Chamaedoreae, Eugeissoneae, Iriarteeae, Sclerospermeae and *Nypa fruticans* (Fig. 2). A further 228 phytoliths were measured at fossil sites, the vast majority of which were Spheroid echinate (217), with ten hat-shaped phytoliths recovered at the mid-Miocene TUR2, and an intermediate (more closely resembling Spheroid echinate) recovered from the late Eocene USA1. For hat-shaped phytoliths, we erected the new morphological name ‘Petasoid echinate’ (following ICPN 2.0; Neumann *et al.*, 2019), with a formal description provided in the Supplementary Data Results S1.

Phytolith morphology varied considerably both between and within the sampled species. Phytolith size was particularly variable, with approximately two orders of magnitude difference between the smallest and largest phytoliths in the modern dataset (4.1–334.4 μm^2^). Intraspecific variation was high, with some species showing variation comparable to entire taxonomic groups (Supplementary Data Fig. S2). Note that due to our sampling strategy (i.e. one individual per species), intraspecific variation could not be distinguished from variation within a single individual.

### Primary and additional discriminant analyses

Among the classification models applied to the palm dataset, model performance varied substantially, both with discriminant method and data treatment. Random forest models trained on the full dataset (i.e. including Spheroid echinate, Petasoid echinate and intermediate morphotypes) provided the best overall performance. For convenience we refer to this model as the ‘standard RF model’. The standard RF model correctly identified 59 % (± 4 %) of test set phytoliths, significantly higher (*P* << 0.001) than the no information rate (NIR; i.e. the expected performance of an uninformed model always guessing the most common taxonomic group) of 27 %. Cohen’s *κ* for the model was 0.51 (values >0.6 are usually taken to indicate substantial agreement of the classifier with true identity; Cohen, 1960). Phytolith area, solidity and aspect ratio were the predictors most important for correctly identifying taxonomic groups in the standard RF model, whereas morphotype (i.e. Spheroid echinate, Petasoid echinate or intermediate), solidity of the internal hull and standard deviation of tubercle height had the smallest impact on model performance.

**Fig. 3.**
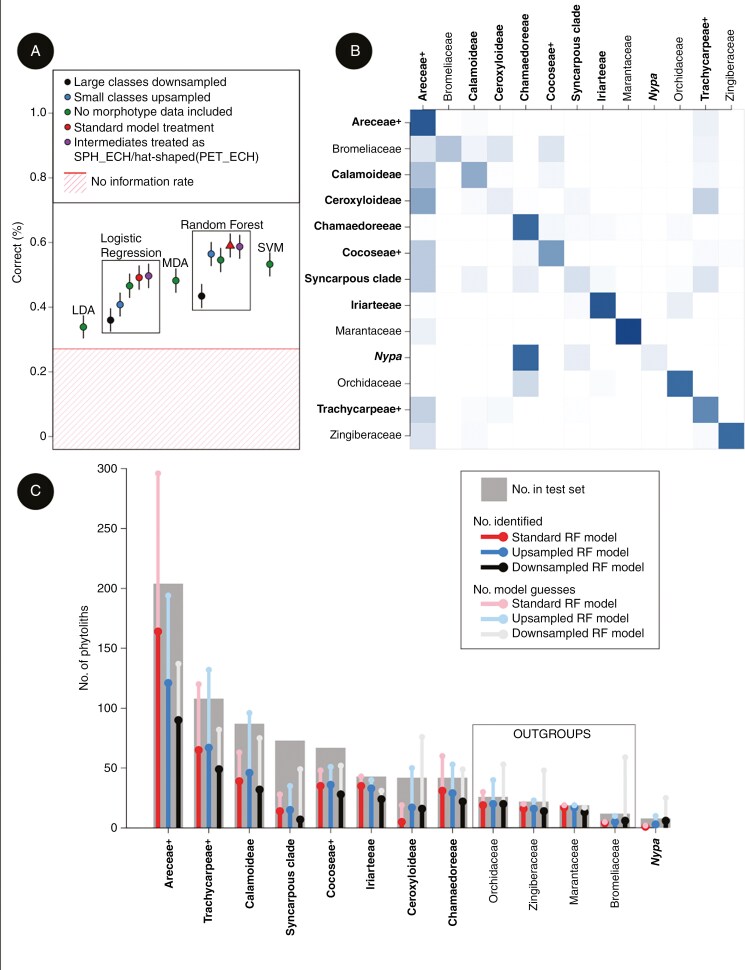
Summary of discriminant analysis results. (A) Variation in overall accuracy for a range of methods and data treatments (sph_ech = Spheroid echinate, pet_ech = Petasoid echinate). (B) Confusion matrix computed from predictions made by the standard RF model (represented by the red triangles in panel A) for our test set. Degree of shading represents the relative number of phytoliths per group assigned to each group, where columns represent model guesses and rows represent true phytolith identity. Shading off the diagonal therefore represents incorrect model assignments. Taxonomic groups within the Arecaceae are bolded. (C) Results for individual taxonomic groups, with number of phytoliths, number of model guesses and number of phytoliths correctly identified indicated for the standard RF model, and RF models with added up- and downsampling (see panel A). Taxonomic groups within the Arecaceae are bolded.

Among the remaining discriminant methods, trained on the full dataset, SVM models provided the best overall performance (accuracy = 53 ± 5 %, *P* << 0.001, *κ* = 0.44), followed by multinomial logistic regression (mLR; accuracy = 49 ± 4 %, *P* << 0.001, κ = 0.39), MDA (accuracy = 48 ± 3 %, *P* << 0.001, *κ *= 0.40) and LDA (accuracy = 34 ± 3 %, *P* << 0.001, *κ* = 0.27) (Fig. 3A). Note that of these models, only mLR models support categorical predictors, and thus included explicit morphotype information.

Spheroid echinate and Petasoid echinate phytoliths differ substantially in their morphology. Consequently, it is important to establish whether ability to correctly identify taxonomic groups differed between these morphotypes, and/or whether overall performance is maximized by including all morphotypes in a single model, or by creating morphotype-specific models. In general, the ability of models to correctly assign Spheroid echinate phytoliths to taxonomic groups was worse than for Petasoid echinate phytoliths. The standard RF model correctly identified the taxonomic group of 55 % (± 5 %) of Spheroid echinate phytoliths in the test set, against an NIR of 37 % (*P* << 0.001, *κ* = 0.39). Note that training models using only Spheroid echinate phytoliths did not result in a higher rate of correct identifications for these phytoliths (accuracy = 54 ± 4 %, *P* << 0.001, *κ* = 0.38). This suggests that including Petasoid echinate phytoliths in the training set did not negatively impact the ability to correctly assign Spheroid echinate phytoliths to taxonomic groups (Supplementary Data Fig. S3).

Petasoid echinate phytoliths were more distinct than Spheroid echinate phytoliths, being correctly identified to taxonomic group 68 % (± 7 %) of the time by the standard RF model, against an NIR of 23 % (*P* << 0.001, *κ* = 0.61). Random forest models trained using only Petasoid echinate phytoliths (accuracy = 68 ± 7 %, *P* << 0.001, *κ* = 0.62) provided a negligible improvement over RF models including all three morphotypes (i.e. the standard RF model). In contrast, including Spheroid echinate phytoliths during model training reduced the ability to correctly identify the taxonomic groups of Petasoid echinate phytoliths in other model types, particularly those not supplied with explicit morphotype information (i.e. in SVM, MDA and LDA) (Supplementary Data Fig. S4).

Ability to correctly assign intermediate phytolith morphotypes to taxonomic groups was generally poor, with the standard RF model returning results that were not significantly better than the NIR (accuracy = 67 ± 15 %, *P* = 0.14, *κ* = 0.39). Treating intermediates as Spheroid echinate or Petasoid echinate marginally increased model performance, although overall accuracy was still not significantly different from the NIR (accuracy = 69 ± 13 %, *P* = 0.08, *κ* = 0.56). An ensemble of the best Spheroid echinate and Petasoid echinate models produced overall results nearly identical to those of the standard RF model (Supplementary Data Fig. S5).

Model performance varied by taxonomic group. In general, models disproportionately guessed the most common groups within our dataset (e.g. Areceae+, Trachycarpeae+, and Calamoideae) at the expense of less common groups. This led to a greater proportion of the phytoliths in large groups identified correctly (i.e. recall), but a relatively lower proportion of guesses of these groups being correct (i.e. precision) (Fig. 3B, Table 1). Typically, phytoliths of outgroups were correctly identified at a high rate by our models. The standard RF model correctly identified ~75 % of phytoliths produced by the Orchidaceae, Marantaceae and Zingiberaceae. The latter two groups were also associated with high precision, meaning few phytoliths from other groups were incorrectly assigned to them (Fig. 3B, Table 1). Although models were usually correct when assigning phytoliths to the Bromeliaceae, the majority of bromeliad phytoliths were incorrectly assigned to palm groups (Fig. 3B). The palm group that was most readily distinguished was the hat-producing Iriarteeae, while the syncarpous clade, Ceroxyloideae, and *Nypa* were the worst performers (Fig. 3B, Table 1). The remaining palm groups generally showed high recall but low precision, or the reverse (i.e. high precision, low recall) (Table 1).

**Table 1.** Group-specific results from the standard RF model for predictions made on the test set, showing the number of phytoliths in each group, the number of phytoliths assigned to that group by the model, and the number of those guesses that were correct. Precision and recall are also given. For each group of palms, we also show median (with first and third quartiles) values for bioclimatic variables and evergreen forest affinity. *NA - indicates groups not included in ecological analyses.

Taxonomic group	No. of phytoliths	No. of guesses	No. correct	Precision	Recall	MAT (°C)	CMT (°C)	MAP (cm)	DQP (cm)	EFA (%)
Areceae+	204	296	164	0.55	0.8	23 (21–25)	17 (14–19)	250 (205–294)	29 (19–44)	88 (84–92)
Bromeliaceae	12	5	4	0.8	0.33	NA^*^	NA	NA	NA	NA
Calamoideae	87	63	39	0.62	0.45	25 (24–26)	20 (18–21)	271 (221–305)	46 (13–55)	86 (81–91)
Ceroxyloideae	42	19	5	0.26	0.12	21 (19–23)	13 (11–15)	190 (142–222)	19 (14–25)	79 (72–85)
Chamaedoreeae	42	60	31	0.52	0.74	22 (20–24)	16 (14–17)	264 (207–294)	19 (15–27)	85 (81–88)
Cocoseae+	67	48	35	0.73	0.52	24 (22–26)	18 (13–20)	194 (142–258)	23 (12–32)	69 (40–86)
Iriarteeae	43	43	35	0.81	0.81	23 (20–25)	17 (15–19)	261 (219–275)	37 (31–45)	93 (92–94)
Marantaceae	19	19	17	0.89	0.89	NA	NA	NA	NA	NA
*Nypa*	8	2	1	0.5	0.13	27 (26–27)	19 (19–22)	208 (172–291)	7 (3–30)	39
Orchidaceae	26	30	19	0.63	0.73	NA	NA	NA	NA	NA
Syncarpous clade	73	28	14	0.5	0.19	24 (23–26)	17 (14–19)	196 (147–238)	11 (7–27)	64 (48–75)
Trachycarpeae+	108	120	65	0.54	0.6	24 (22–25)	15 (11–18)	148 (116–200)	11 (4–18)	49 (32–75)
Zingiberaceae	22	20	16	0.8	0.73	NA	NA	NA	NA	NA

Random forest models predicting subfamily classification correctly identified slightly more phytoliths than the standard RF model, although this was relative to a much higher NIR (47 %), and *κ* values were also reduced (accuracy = 64 ± 3 %, *P* << 0.001, *κ* = 0.43). In contrast, RF models predicting tribe classification showed reduced overall accuracy relative to the standard RF model (accuracy = 47 ± 3 %, *P* << 0.001, *κ* = 0.41), although the NIR was also significantly reduced (17 %).

Efforts to account for the effects of class imbalance through up- and downsampling during cross-validation of RF models had mixed success. Downsampling of large groups resulted in an increase in the number of phytoliths correctly identified within the least populated groups (Bromeliaceae and *Nypa*). However, gains were modest (seven additional phytoliths correctly identified) and came at the expense of model precision among the smallest groups, and the total number of phytoliths correctly identified (Fig. 3A, C). By contrast, upsampling generally resulted in overall results more comparable to the standard RF model (Fig. 3A), while offering modest increases in the number of phytoliths correctly identified among some of the smaller groups (Fig. 3C). The relative effects of up- and downsampling during model training varied by method, with some (e.g. mLR) showing more marked changes to overall performance. Random forest was generally the best performing in this regard (Fig. 3A).

In summary, random forest models (e.g. our standard RF model) provided the best overall performance, correctly identifying 59 % of phytoliths, significantly higher than the NIR. Petasoid echinate phytoliths were correctly identified at higher rates than other morphotypes, with those produced by the Iriarteeae the most distinct among sampled palms. Models were generally able to distinguish palm phytoliths from those of outgroup taxa, with those produced by the Bromeliaceae a notable exception. Ability to identify the remaining taxonomic groups was mixed, with models disproportionately assigning phytoliths to the two largest groups (Areceae + and Trachycarpeae+).

### Ecological reconstructions and comparative analyses

**Fig. 4.**
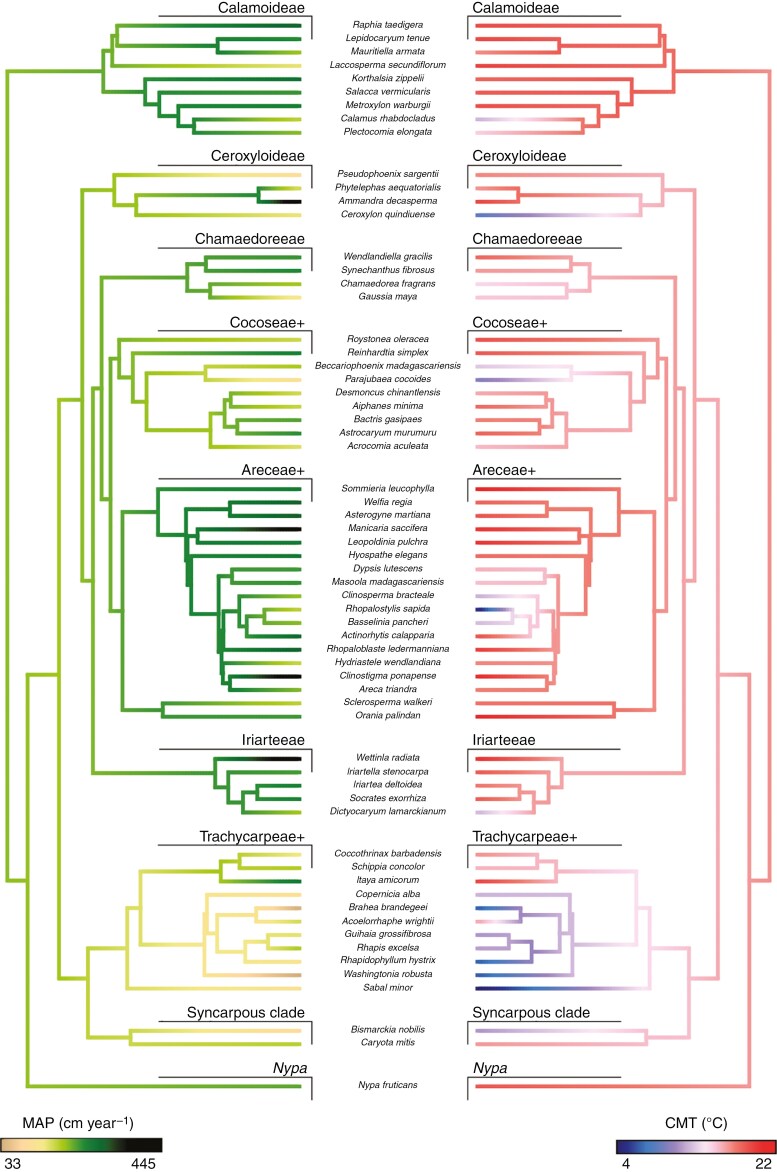
Ancestral state estimations of mean annual precipitation (MAP, cm*year^−1^) and minimum temperature of the coldest month (CMT, °C) for taxa for which we obtained both phytolith and climate data. Ancestral state estimates were based upon a dataset compiled for >1000 species, and pruned to show only those represented in the phytolith dataset.

In total, we obtained 65 040 palm occurrence records that met our vetting requirements, representing 1017 palm species, 63 of which were represented in our phytolith dataset. Most sampled lineages occupied warm and equable regions, which was inferred as the ancestral habitat for all major groups (Table 1, Fig. 4, Supplementary Data Fig. S6). The Ceroxyloideae and Trachycarpeae+ were generally the most cold-tolerant, both including taxa found in seasonally cool regions (Table 1, Fig. 4). Mean annual rainfall data suggested that most groups occupied wet habitats, with the majority of species found in regions where rainfall exceeded 200 cm year^−1^(Table 1). Although the family was ancestrally adapted to wet habitats (~220 cm year^−1^), transitions to regions with very high year-round rainfall appeared more recently, particularly within the Arecoideae (i.e. Areceae+, Chamaedoreae, Cocoseae+, and Iriarteeae) and Calamoideae (Fig. 4, Supplementary Data Fig. S6). In contrast, the Trachycarpeae+, syncarpous clade and *Nypa* all occupy regions where rainfall is more markedly seasonal (Table 1). The ancestral habitat of palms was inferred to have been one of moderate rainfall seasonality, with estimated dry quarter precipitation constituting ~10 % (~24 cm) of inferred annual precipitation. In most groups, EFA was high (0.69–0.93; Table 1), consistent with the inferred ancestral habitat of the family (Supplementary Data Fig. S6). The exceptions were the Cocoseae+, Trachycarpeae+, syncarpous clade and *Nypa*, each of which included a considerable number of taxa more frequently found outside of evergreen forests (i.e. EFA < 0.5) (Table 1).

Results from multilevel models showed model convergence and adequate sampling of the posterior (Rhat values near the convergence expectation, effective sample sizes >400 for all model parameters). Correlations between environmental predictors and phytolith morphology were consistently weak, with credibility intervals for slope parameters suggesting no effect (i.e. overlapping with zero) in nearly all cases (Supplementary Data Fig. S7). The only exceptions were a weak negative correlation between phytolith circularity and dry quarter precipitation (i.e. rounder phytoliths weakly correlated with more seasonally dry climes), and a weak positive correlation between the standard deviation of tubercle heights and the minimum temperature of the coldest month (i.e. smaller variation in tubercle size in seasonally cool climes). Owing to the consistently weak correlation between environmental predictors and phytolith morphology we did not use multilevel models in any of our palaeoenvironmental reconstructions (see next section).

### Fossil predictions and nearest living relative analysis

**Fig. 5.**
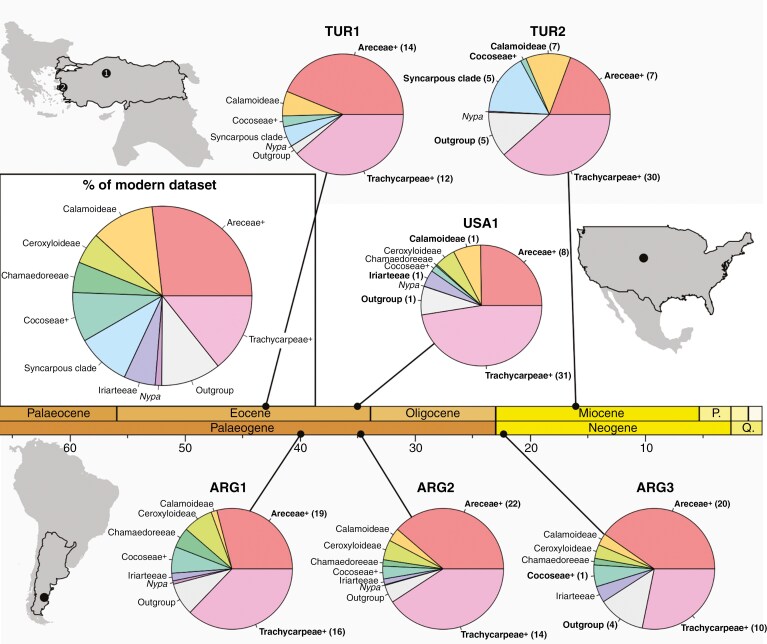
Pie charts showing the average proportions of phytoliths assigned to each taxonomic group, the location and approximate age (millions of years) of each sampled fossil site. Averages account for uncertainty in model assignments and assemblage sample size through bootstrapping, and make the following biogeographic assumptions: (1) the syncarpous clade was absent from North and South America during the Cenozoic; (2) *Nypa* was absent from Neogene sites in the Americas; and (3) Ceroxyloideae, Chamaedoreeae and Iriarteeae were absent from Turkey. Taxonomic groups are bolded if considered the best guess for at least one phytolith in the assemblage by the standard RF model (i.e. unbolded taxonomic groups were not considered the best for any single phytolith). The number of phytoliths assigned to each bolded group is also indicated. Proportions of phytoliths from each taxonomic group in the modern training set are provided in the inset pie chart. Note that the Areceae and Trachycarpeae are the two largest groups in the modern dataset, and those which are most frequently predicted among the fossil dataset.

Model predictions suggest fossil assemblages were dominated by the Areceae+ and Trachycarpeae+, the two most common groups within the modern training dataset (Fig. 5). Among analysed sites, TUR2 (Miocene of Turkey) was the most diverse, with models recognizing the presence of five different taxonomic groups, including the Orchidaceae. Zingiberaceae (ARG3, USA1) was the only other outgroup to be identified by model predictions. At North and South American sites, several phytoliths were assigned to the syncarpous clade, although this lineage was likely absent from the region. In biogeographically constrained models these phytoliths were assigned to the Areceae+ (ARG1, ARG2, ARG3) or Trachycarpeae+ (USA1). At TUR2, two Petasoid echinate phytoliths assigned to the Iriarteeae were assigned to the Orchidaceae in biogeographically constrained models. In total, ten Petasoid echinate phytoliths were measured at TUR2; taking biogeographic considerations into account, models assigned four to the Orchidaceae and the remainder to the syncarpous clade. When accounting for both model uncertainty and assemblage size through bootstrapping, reconstructions suggested a greater number of lineages present at each site. However, the Areceae+ and Trachycarpeae+ were still inferred to make up the majority of each assemblage (Fig. 5).

Environmental conditions inferred at sites from biogeographically constrained phytolith assemblage reconstructions are presented in Table 2. The range of potential mean annual temperature was between 16 and 27 °C at all sites, with those in the Americas skewing slightly cooler than those in Turkey. Ranges of cold month temperature were similar across sites, falling between 8 and 22 °C. The range of mean annual precipitation values at each site was wide, ranging from a minimum of ~100 cm year^−1^ at most sites to >300 cm year^−1^. Excluding the oldest Argentinian site, those in the Americas were inferred to be marginally wetter than those in Turkey, with ranges exceeding 400 cm year^−1^. At each site, estimates of dry quarter precipitation usually accounted for ~15 % of annual rainfall (taking median values of MAP and DQP). However, the range of values was wide. Sites in Turkey skewed drier, with lower estimates of only 2 cm of dry quarter rainfall. Evergreen forest affinity of the taxa present at sites was high, with median values of at least 85 % at all sites. This value reflects the proportion of occurrences found in evergreen forests for the typical member of the groups present at the site (Table 2).

**Table 2.** Range of plausible conditions at fossil sites inferred from nearest living relative analyses using a biogeographically constrained coexistence approach. Estimated conditions are given for MAT, CMT, MAP, DQPand EFA. The ranges incorporate 95 % confidence intervals for values obtained through bootstrapping to account for uncertainty due to model uncertainty and assemblage size. In contrast to the remaining environmental variables, EFA is provided as the median (with first and third quartiles), reflecting the evergreen forest affinity of the typical taxon at the site (see Materials and methods section). The location and approximate age in millions of years are also given for each site.

Site	Location	Age (Ma)	MAT (°C)	CMT (°C)	MAP (cm)	DQP (cm)	EFA (%)
ARG1	South-east Argentina	39.9	17–26	10–21	126–365	10–57	88 (63, 98)
ARG2	South-east Argentina	34.8	16–26	9–21	110–422	8–71	89 (63, 97)
ARG3	South-east Argentina	22.5	17–26	11–21	142–413	18–66	86 (57, 95)
TUR1	Central Turkey	47.8–37.8	20–27	8–22	90–361	2–65	88 (64, 96)
TUR2	Western Turkey	16	21–27	9–22	90–316	2–58	89 (66, 97)
USA1	Central USA (NE)	34–36	17–26	11–21	142–442	18–72	86 (63, 97)

## DISCUSSION

Our survey of palm phytolith morphology shows that there is phylogenetically informative shape and size variation within the family; for example, the production of Petasoid echinate (i.e. hat-shaped) phytoliths is limited to certain clades rather than occurring across all palm subfamilies or tribes (Fig. 2), a result consistent with previous observations (Prychid *et al.*, 2003). However, overlapping phytolith shape and size variation within palms, particularly the high level of intraspecific variation (Supplementary Data Fig. S2), and similarities with phytoliths produced by some of the outgroup taxa make distinguishing many palm clades difficult.

### Model selection and performance: can phytoliths distinguish palm clades?

We evaluated the potential for phytolith morphology to distinguish important palm clades using more than 20 different discriminant models, encompassing five different statistical methods and a range of data treatments. Of these, the standard RF model had the best overall performance (Fig. 3A), and was among the best performing for each individual taxonomic group. Random forest models are known to perform better than many methods when dealing with small sample sizes and complex predictors (Qi, 2012), both of which are features of our phytolith dataset. Models accounting for class imbalance through up- or downsampling did increase the number of phytoliths correctly identified within some of the less speciose groups, but gains were modest and balanced by poor performance in larger groups (Fig. 3C). These approaches are also prone to overfitting (i.e. highly predictive of the training data but not new datasets) (Elhraman and Abraham, 2013). Models using our 13 taxonomic groups (Fig. 2) were also preferred over those predicting subfamily or tribe. Although subfamily models correctly identified slightly more phytoliths than the standard RF model, they showed more pronounced bias towards more speciose groups and less improvement over the NIR (Supplementary Data Fig. S8). Tribe models were also generally outperformed by the standard RF model (Supplementary Data Fig. S9). Furthermore, several extant tribes likely diverged during the Eocene (Baker and Couvreur, 2013), roughly coincident with many of our fossil sites. Attempting to assign phytoliths to lineages that had not yet (or only recently) diverged may be problematic, and clades with deeper divergences, as those in our standard model (Fig. 2), likely form a more appropriate framework for interpreting deep time palaeovegetation.

Although it outperformed the other models tested, concerns remain about the potential of the standard RF model as a tool for reconstructing fossil palm phytolith assemblages. Despite correctly identifying significantly more phytoliths than expected under the NIR (Fig. 3A), the proportion of correct guesses (59 %) may not inspire researcher confidence. The model’s tendency to disproportionately assign phytoliths to our largest groups, the Areceae+ and Trachycarpeae+ (Fig. 3B, C), is also likely to cause concern. Such bias towards better sampled groups is a common problem in machine learning studies (Japkowicz and Shaju, 2002; Johnson and Khoshgoftaar, 2019). Ultimately, unless traits clearly identify a phytolith as belonging to a particular group, the model prediction most likely to be correct is the group with the most phytoliths in the training set. This bias towards more speciose groups likely contributed to our reconstruction of all six fossil sites as being dominated by the Areceae+ and Trachycarpeae+. Codominance of these clades does occur at some modern sites (e.g. rainforests of north-eastern Australia; Tng *et al.*, 2016), but it is a pattern that is uncommon in many regions (e.g. Kahn and de Castro, 1985; Bongers *et al.*, 1988; Kahn and Mejia, 1990; Peres, 1994; Listabarth, 1999), consistent with the ecological differences between the two groups (Table 1, Fig. 4). Although non-analogue vegetation is not infrequent in the fossil record, pointing to shifting plant ecologies through time (e.g. Gayó *et al.*, 2005; Dunn *et al.*, 2015), independent evidence would be necessary to verify any conclusions based on our classification model. The tendency for models to misidentify bromeliad phytoliths as palms is also concerning. Although previous work differentiated these groups using phytolith dimension, tubercle size and tubercle frequency (Benvenuto *et al.*, 2015), our more comprehensive sampling suggests that tubercle and phytolith size variation within the palms completely encompasses that observed within sampled Bromeliaceae (see the section Recommendations for future work using palm phytoliths in palaeoecology).

Difficulties with the bromeliads notwithstanding, the general ability of the standard RF model to discriminate between palm and the remaining non-palm phytoliths (i.e. Marantaceae, Orchidaceae, Zingiberaceae; Fig. 3B, C) is encouraging. Within Arecaceae, the most distinguishable groups were typically those producing Petasoid echinate phytoliths. These phytoliths were phylogenetically restricted, and correctly identified at a substantially higher rate than the more common Spheroid echinate phytoliths. Although rare in the fossil record, Petasoid echinate phytoliths have been recovered at several sites (e.g. Strömberg *et al.*, 2007; C. A. E. Strömberg, unpubl. data). In sub-Recent contexts, these phytoliths have been used as evidence for the presence of mangrove swamps (e.g. Das *et al.*, 2014; Chabot *et al.*, 2018). However, extension of this proxy to deep time contexts is potentially problematic. Although mangrove palms (*N. fruticans*) produce Petasoid echinate phytoliths, our results show that Petasoid echinate morphotypes are produced by several phylogenetically disparate palm clades (Fig. 2; Prychid *et al*., 2003), and those produced by *Nypa* are not readily distinguished from those of other hat-producing clades. Thus, inferring the presence of mangrove swamps should be supported by additional evidence (e.g. other micro- or macrofossils; Ellison *et al.*, 1999; Srivastava and Prasad, 2019) and not rely on Petasoid echinate phytoliths alone.

The Petasoid-echinate-producing Iriarteeae was the most distinct group within the Arecaceae (Fig. 3B, C), with phytoliths that tend to be large, oblong, and have irregular margins. The relative reliability with which this group can be identified, coupled with its proclivity for warm, wet and equable evergreen forests (more than almost any other sampled group; Table 1, Fig. 4) makes it a potentially useful habitat marker in the fossil record. By contrast, the coryphoid palms (i.e. syncarpous clade, Trachycarpeae+), which include many of the extant lineages occupying seasonally cool and dry habitats (Table 1, Fig. 4), were among the most poorly recognized by our models. The failure to consistently distinguish coryphoid palms has important implications for palaeoenvironmental inference, reinforcing that inferring warm and wet, evergreen forests from palm phytoliths alone may be problematic, given the difficulty of identifying phytoliths of taxa that deviate from this ecology. Finally, our failure to recover any strong relationship between phytolith morphology and environmental factors, once phylogenetic relationships had been accounted for, suggests that reconstructing palaeoenvironment directly from palm phytolith morphology is infeasible.

### Reconstructions at fossil sites: can palm phytoliths infer past environments?

Inferring past climates from fossil palm phytolith assemblages depends not only on our ability to determine which lineages were present (discussed above), but also on the evolution of habitat preferences within the Arecaceae. There is some debate over whether the earliest palms occupied seasonally dry habitats (Thomas and Boura, 2015) or habitats like the tropical rainforests, where the majority of palm diversity is found today (Couvreur *et al.*, 2011; Cásia-Silva *et al.*, 2019). Results from our comparative analyses are intermediate between these two hypotheses. Although they suggest the earliest palms experienced some precipitation seasonality, the dry season was not reconstructed as being particularly pronounced (> 20 cm per quarter). And although annual rainfall in the ancestral palm habitat was estimated to be high (~220 cm yr^−1^), we also recovered more recent trends towards even higher and more aseasonal rainfall, characteristic of many extant Arecoideae and Calamoideae (Fig. 4, Supplementary Data Fig. S6).

With the limitations to discriminant models (discussed above) in mind, we compare our reconstructions at the investigated fossil sites with palaeoenvironmental evidence from previous publications. Palm phytolith assemblages measured at all three Patagonian sites are consistent with a warm, wet, evergreen forest (Table 2). However, the potential range of total yearly rainfall and its seasonality is large, spanning conditions observed in extant Amazonian rainforest (Sombroek, 2001) and climates with pronounced dry seasons (e.g. wetter regions of the Pampas grasslands; Krapovickas and Giacomo, 1998; Aliaga *et al.*, 2017). Published palaeoecological reconstructions are more consistent with the drier end of this inferred range, with recent work suggesting shrubland, dry forest or scrub at all three sites (Dunn *et al.*, 2015). Although MAT estimates of previous studies are consistent with those we obtained (~18 °C at each site), precipitation estimates tend to fall below our ranges (~100 cm year^−1^), particularly when obtained from palaeosol data or stable carbon isotopes of tooth enamel (Hinojosa, 2005; Hinojosa and Villagrán, 2005; Bellosi and González, 2010; Dunn *et al.*, 2015; Kohn *et al.*, 2015). Reconstructions of Patagonian sites as open shrubland or forest-scrub also contrast with our reconstructions which favour evergreen forests. Nevertheless, a dry, or seasonally dry, open habitat could explain the abundance of the Trachycarpeae and its relatives at the sites, given that some members of this group are found in arid or seasonally arid regions of the Americas today (e.g. Felger and Joyal, 1999; Laughlin, 2002).

Like the Patagonian assemblages, the palaeoenvironmental reconstructions in the late Eocene of the central USA (USA1) suggest a warm, wet, evergreen forest, although the range of possible precipitation conditions is large. Previous work generally supports this interpretation. Phytolith assemblages are dominated by forest indicators and phytoliths diagnostic of closed habitat grasses (bamboos in the tribes Bambuseae and Olyreae), suggesting a closed forest habitat (Strömberg, 2005; Gallaher *et al.*, 2020). Although analyses of fossil wood (Wheeler and Landon, 1992) and palaeosols (Retallack, 1997, 2001; Terry, 2001) point to a trend of aridification and increased rainfall seasonality during the deposition of the Chadron formation, the Peanut Peak member was deposited near the beginning of this sequence where more humid conditions apparently prevailed (Terry, 1998, 2001). Further, recent geochemical analyses challenge the notion of strongly seasonal, arid climates in the Late Eocene, instead indicating minor rainfall seasonality in the continental interior of the USA until the Oligocene–Miocene transition (Kukla *et al.*, 2022). Temperature estimates are also broadly congruent with previous work, which indicates MAT ~20 °C in central North America during the period following the Middle Eocene Climatic Optimum and preceding the Eocene–Oligocene Transition, when this phytolith assemblage was deposited (Zanazzi *et al.*, 2007). High abundance of Trachycarpeae+ inferred at USA1 is also consistent with extant palm biogeography, since taxa in this group are among the most diverse and abundant in North America today (Zona, 1993).

Both palm phytolith assemblages from the Eocene and Miocene of Turkey (TUR1 and TUR2) were consistent with warm, wet, evergreen forests. However, the possible range of annual and dry season precipitation was large, and skewed marginally drier than the other sites. This leaves open the possibility that both TUR1 and TUR2 were at least seasonally dry. At TUR1, our reconstructions are broadly concordant with previous work, which generally supports a closed forest and tropical climate with abundant rainfall (Akgün *et al.*, 2002; Strömberg *et al.*, 2007). At TUR2, our reconstructions suggest wetter conditions than previous work, which fall near the dry end of the reconstructed plausible range (Akgün *et al.*, 2007; Akkiraz *et al.*, 2011; although see Denk *et al.*, 2019). Previous phytolith analysis indicates more open savannas, woodlands or grassland mosaics (Strömberg *et al.*, 2007), while macro- and palynofloras (Denk *et al.*, 2017) imply mixed laurel and broadleaf deciduous forests. Both interpretations are at odds with the high EFA we infer for the assemblage. Although our temperature reconstructions at TUR2 do not differ markedly from published work, we do not find clear evidence of the cooling trend that likely occurred in the region from the Palaeogene onward (Akgün *et al.*, 2007; Denk *et al.*, 2019). However, we did detect an increase in the abundance of phytoliths attributed to the relatively cold-tolerant syncarpous clade (Figs 4 and 5).

Although predictions at our fossil sites seem to favour the most common groups, the frequency of these predictions appears to not be strictly a function of imbalance in our modern dataset (Fig. 5). Phytoliths were disproportionately assigned to the Trachycarpeae+, which was the most common group at most fossil sites (when accounting for model uncertainty) despite constituting only 14 % of our training set. The abundance of this clade at fossil sites therefore cannot be explained solely by the composition of our training set. An additional important factor may be the size of phytoliths in fossil assemblages; Trachycarpeae+ phytoliths tend to be ~50 % larger than the family average, while measured fossil assemblages are ~100 % larger. It is unlikely that the size discrepancy between fossil and modern datasets represents a true taphonomic bias (e.g. preferential preservation of larger phytoliths), because all the studied assemblages are known to contain palm phytoliths of the smallest class (≤5 μm diameter) (Strömberg, 2005; Strömberg *et al.*, 2007, 2013; C.A.E. Strömberg, pers. obs.). Instead, we suggest that our sampling was skewed, as these smaller phytoliths can be difficult to find and image at the maximum magnification possible with the available equipment (×400–500), particularly when other silica particles (e.g. volcanic ash, diatoms) are also abundant. Such contaminants, which obscure the view, are not present in most modern phytolith samples, resulting in a difference in what particles are selected for imaging. Nevertheless, we cannot rule out that ancient palms produced larger phytoliths than their extant relatives. Given that our comparative analyses did not find any strong correlation between habitat and phytolith morphometric features, it is doubtful that climate or habitat directly contributed to the observed size patterns, although other environmental factors may warrant investigation (e.g. CO_2_ concentration; Pritchard *et al.*, 1999; Johnson and Hartley, 2018).

### Recommendations for future work using palm phytoliths in palaeoecology

To date, this study represents the most comprehensive attempt to quantify the morphology of stegmata-derived palm phytoliths, in an attempt to (1) separate palms from non-palm taxa and (2) distinguish between different palm taxa and ecologies across the Arecaceae as a whole. We devised our method to limit error arising from manual measurements; however, because of the small size and hence blurry outline of many of the phytoliths, it was not possible to completely automate our process (e.g. the outline had to be largely hand-drawn). Future work should consider using confocal microscopy on fluorescent-stained phytoliths for a precise and detailed 3-D rendering of palm phytoliths, as has been done for phytoliths of grasses (Gallaher *et al.*, 2020). Such an approach might allow the inclusion of not just 3-D shape (e.g. the height of the dome of the Petasoid echinate), but also more specific information about shapes, sizes and distribution of tubercles thought to be taxonomically informative (e.g. Fenwick *et al.*, 2011; Benvenuto *et al.*, 2015; Morcote-Ríos *et al.*, 2016; Huisman *et al.*, 2018; Witteveen *et al.*, 2022). In particular, these additional traits may help distinguish bromeliad phytoliths, which purportedly possess fewer, less well defined, and irregularly distributed tubercles compared with palms (Piperno, 2006; Benvenuto *et al.*, 2015). Nonetheless, there is substantial overlap between phytoliths from bromeliads and palms in both shape and size such that we presently urge caution when identifying individual phytoliths, whether it is done by experts or by applying our method. Using machine learning methods for automatic identification and classification of stegmata-derived phytoliths could also be explored in future work (Díez-Pastor *et al*., 2020; Andriopoulou *et al*., 2023).

Expansion of the modern dataset to include a wider range of palm taxa and outgroups is likely to enhance the predictive models. For example, only a small portion of species diversity was sampled for the included outgroups, which are known to produce a wide range of phytolith morphologies (e.g. Chen and Smith, 2013; Benvenuto *et al.*, 2015). In this study our modern dataset was composed of phytoliths extracted from leaf tissues. However, Spheroid echinate and Petasoid echinate phytoliths are known to be produced in other tissue types as well (Neumann *et al.*, 2019; Supplementary Data Results S1). Although foliar phytoliths should account for the majority of the diagnostic morphotypes recovered from fossil assemblages (Mulholland, 1989), expanding our sample to include phytoliths produced in other organs may also prove fruitful.

Based on the available data, our work suggests that palm phytoliths provide limited precision and accuracy as a tool for reconstructing ancient palm evolution and ecology. Although the palaeoenvironmental inferences from phytolith assemblages in some cases agree with previous palaeoclimate reconstructions, they often infer tropical, wet habitats even when other palaeoclimate data provide contrasting habitat signals. Thus, relative to some previous work (e.g. Huisman *et al*., 2018), we urge more caution in ecological interpretation of palm phytoliths. Notably, where prior work has generally focused on taxonomically or geographically restricted samples (e.g. Albert *et al.*, 2009; Fenwick *et al*., 2011; Bowdery, 2014; Morcote-Ríos *et al.*, 2016; Huisman *et al*., 2018), our broader survey reveals a correspondingly large range of morphological variation in the family, with considerable overlap between groups – and some outgroups (e.g. Bromeliaceae). These morphological patterns lead to uncertainty in the taxonomic placement of phytoliths, particularly for the Spheroid echinate morphotype. Whereas a narrower taxonomic or geographic scope can improve performance and may be justifiable in archaeological or recent palaeontological contexts (Morcote-Ríos *et al.*, 2016), in deep time applications the pool of potential species is inherently broader and more ambiguous. As such, a conservative approach is likely appropriate for most palaeontological applications.

In this study, we opted to use a coexistence approach to explore the environmental implications of our taxonomic identifications. However, future work should ideally seek to incorporate recently developed, alternative methods for palaeoclimate reconstruction that might produce more robust results (Grimm and Denk, 2012; Grimm and Potts, 2016; Willard *et al.*, 2019; Klages *et al*., 2020). Unfortunately, all available approaches rely on accurate taxonomic identification, which at present is the primary limitation to accurate palaeoclimate reconstruction based on palm phytolith assemblages.

Despite its limitations, we believe our method shows promise if applied with appropriate caution. The model is most promising for identifying certain distinct groups, particularly petasoid-echinate-producing palms. For example, confirming the presence of the Iriarteeae is both palaeoenvironmentally informative and feasible given model performance, although the rarity of fossil Petasoid echinates and the restriction of the Iriarteeae to the Americas may restrict this group’s usefulness. Nevertheless, the distinctiveness of Petasoid echinate phytoliths is encouraging, especially given that the measurement protocol was optimized for the more common Spheroid echinate phytoliths. Future optimization for Petasoid echinate phytoliths may result in additional improvements to model performance among these groups.

### Conclusions

Using the most comprehensive morphometric dataset of palm phytoliths assembled to date, we evaluated the utility of palm phytoliths as a taxonomic and palaeoecological tool. In contrast to some previous work, our results suggest that palm phytolith morphology does not generally provide reliable information about Arecaceae intrafamilial taxonomy. Because cryptic phytoliths are produced by ecologically diverse groups, we recommend against palaeoecological inference using palm phytoliths at present. Nevertheless, additional work could make rarer, and generally more distinct, Petasoid echinate (i.e. hat-shaped) palm phytoliths a useful complementary tool for testing evolutionary or palaeoecological hypotheses in a wider range of fossil contexts.

## SUPPLEMENTARY DATA

Supplementary data are available at *Annals of Botany* online and consist of the following. Table S1: Raw data used in discriminant analyses for modern reference and fossil phytoliths, including taxonomic and collections information. Table S2: Vetted occurrence records and associated environmental data used in ecological analyses. Methods S1: Additional microscopy methods. Methods S2: ImageJ macro and details of its use. Methods S3: Additional methods of occurrence record vetting. Figure S1: Priors used for Bayesian multilevel models testing correlation between phytolith morphology and environmental variables. Results S1: Description of Petasoid echinate morphotype. Figure S2: Phytolith phylomorphospace. Figure S3: Summary of discriminant analysis results for models predicting Spheroid echinate identity. Figure S4: Summary of discriminant analysis results for models predicting Petasoid echinate identity. Figure S5: Comparison of standard RF model with an ensemble of the best individal morphotype models. Figure S6: Ancestral state reconstructions of mean annual temperature, dryest quarter precipitation and evergreen forest affinity. Figure S7: Results of Bayesian multilevel models. Figure S8: Summary of discriminant analysis results for predicting subfamily. Figure S9: Summary of discriminant analysis results for models predicting tribe.

mcae068_suppl_Supplementary_Materials
