# Supplementary material for: Palms of the past: can morphometric phytolith analysis inform deep time evolution and palaeoecology of Arecaceae?
Source: Ann Bot. 2024 Apr 30;134(2):263–82. doi: 10.1093/aob/mcae068 (PMC11232524; doi:10.1093/aob/mcae068)
Supplement: mcae068_suppl_Supplementary_Materials [file mcae068_suppl_supplementary_materials.zip › Palm_Supplement_Resub_final.doc]

**Table S1:** Provided as separate file. Raw data used in discriminant analyses for modern reference and fossil phytoliths, including taxonomic and collections information. Classification in the hat column indicates whether phytoliths were identified as hat-shaped (Y), Spheroid echinate (N), intermediate recovered from a hat-producing taxon (PY), or intermediate recovered from a Spheroid echinate producing taxon (PN). The second tab provides geographic, collections, and age data for fossil sites used in this study.

**Table S2:** Provided as separate file. Vetted occurrence records and associated environmental data used in ecological analyses. For each record, species name and coordinate data are given. Environmental data are as follows. Most frequently observed landcover type given as original pixel values (landcover), land cover description (full_label) and an abbreviated description (abrv_label). Bioclimatic variables (bio1 – bio19) obtained from WorldClim surfaces, and landcover pixel values from 1992 – 2015 (ESA92 – ESA15).

**Methods S1:** Phytoliths were imaged with 1) a Leica TCS SP5 Confocal Microscope with a Plan Apo CS 63x oil immersion lens, NA 1.4, a 405 nm laser at 50% power, a substrate beam splitter, and a transmitted light detector with a gain around 210 V; 2) A Nikon A1 Confocal Microscope with a Plan Apo λ 60x oil immersion lens, NA 1.4, a 488 nm laser at 50% power, a substrate beam splitter with a gain around 50 V. Z-stacks of whole phytoliths were acquired with a step size of 0.13 (Leica) or 0.10 (Nikon) µm, 512 x 512 pixels, no frame averaging, and saved as LIF (Leica) or nd2 files (Nikon). The latter equipment was used to image a small number of hat-shaped phytoliths (n = 9) from the fossil site TUR2, as these phytoliths were found and imaged following replacement of much of the equipment in the University of Washington’s Biology Imaging Facility.

**Methods S2:** A custom macro was used to extract morphologic features from z-stacked images of phytoliths. The steps of the macro are briefly described below, followed by details for readers interested in replicating its function. The macro code is provided as a separate file with the submission.

A composite image of the entire z-stack was generated, on which the user traced the edge of the phytolith using the ImageJ polygon selection tool. This selection was then smoothed via fitting of a spline. The background was then cleared and a gaussian blur applied to the image, creating a pixel gradient that allowed the user to define the “internal hull” of the phytolith, bounded by the deepest sinus between two tubercles (Fig. 1b), using the wand tool (note users are also asked to define a larger version of this selection bounded by the shallowest sinus between two tubercles, this was not used in any of our subsequent analyses). Standard shape and size descriptors (area, perimeter, aspect ratio, solidity, circularity) were extracted from the phytolith outline and the “internal hull” of the phytolith. Typical tubercle size and variation was measured as the median and standard deviation of the distance between the “internal hull” and convex hull of the phytolith outline (after exporting as x-y coordinates), which respectively define the upper and lower extent of the tubercles (Fig. 1b). All user selections were output as overlays on measured images, and vetted by at least two researchers to ensure sufficient image quality and agreement on outline placement.

*Changing the macro to suit your file format -* The provided macro is originally configured to work with raw image files having "LIF" as a file format. It is important to note that it is advisable to save your raw images in the file format associated with your microscope (e.g., LIF, nd2, etc.) for optimal compatibility. To adapt the macro to your specific file format, follow these steps:

*Open the macro:*

- Open the macro script in a text editor of your choice. The macro script typically has a file extension of ".ijm."

*Locate the line:*

- namenoext = replace(filename, ".LIF", "");

*Replace file format:*

- Replace ".LIF" in the line with the file format extension that corresponds to your images.
- For example, if your images are in ".nd2" format, the line should be modified as follows: namenoext = replace(filename, ".nd2", "");

*Save the macro:*

- Save the modified macro script with the same name and ".ijm" file format extension.

**Setting up and running the macro**

*Install ImageJ and macro:*

- Ensure ImageJ is installed on your computer. If not, download and install it from the official website.
- Place the "SultanPalmProject_v3.0.0" macro file in the ImageJ 'macros' directory.

*Starting ImageJ:*

- Open ImageJ. The "AutoRun" macro will execute automatically, setting up the necessary environment for "SultanPalmProject_v3.0.0."

*Running the macro:*

- In ImageJ, go to Plugins → Macros → Run Macro...
- Select and run the "SultanPalmProject_v3.0.0" macro.

**Using the macro for image analysis**

*Importing the image:*

- The macro initiates with the Bio-Formats Importer. Select the microscopy image file of the phytolith you wish to analyse.

*Setting up the output directory:*

- After opening the image, you will be prompted to choose a directory to save the output files. Select an appropriate folder.

*Initial image processing:*

- The macro automatically processes the image, including a z-projection and a conversion to 8-bit. It will save an initial processed version of the image.

*User interaction for phytolith outlining and selecting internal hulls and tubercle heights of the phytolith:*

- Follow the on-screen prompts to outline the phytolith using the polygon selection tool. Next, follow the on-screen prompts to make necessary corrections to the spine by clicking on the designated points and dragging the mouse. In the following step, select a point in the centre of the phytolith. Then, adjust the tolerance so that you reach the base of the deepest tubercle. After this, select a point in the centre of the phytolith and adjust the tolerance so that you reach the base of the shallowest tubercle.

*Saving results and data:*

- The macro will automatically save measurements in CSV format.
- The final processed image will also be saved in the chosen output directory. All user selections are output as overlays on the measured images (image file with suffix *_final.tif*)

*The output file needed for further analysis is:*

- File with suffix *_measures.csv* – standard size and shape variables extracted from the user defined phytolith outline, the convex hull of the phytolith outline, the larger “internal hull” (note not used in any analyses), and the smaller “internal hull” **in that order.**
- File with suffix *_Tubercle_heights.csv* – distribution of distances between the “internal hull” and the convex hull of the phytolith outline. The median and standard deviation of this distribution was used in our analyses.

**Methods S3:** Georeferenced occurrence records were downloaded from the Global Biodiversity Information Facility (GBIF) and vetted using GBIF’s internal quality tags, limiting records to just those associated with preserved specimens collected after 1949, and excluding all those which were flagged with any of the following notifications.

CONTINENT_INVALID

PRESUMED_NEGATED_LONGITUDE

TAXON_MATCH_FUZZY

COORDINATE_ROUNDED

COUNTRY_INVALID

GEODETIC_DATUM_INVALID

PRESUMED_NEGATED_LATITUDE

PRESUMED_SWAPPED_COORDINATE

COUNTRY_MISMATCH

TAXON_MATCH_HIGHERRANK

The remaining records were subjected to several tests implemented in the R (v. 4.0.5; R Core Team, 2021) package CoordinateCleaner (v 2.0-11; Zizka et al., 2019) to exclude records assigned the coordinates of the capital or centroid of the country in which they were found (within 1km; “capitals” and “centroids” tests) or those assigned the coordinates of biodiversity institutions (e.g., herbaria where specimens are stored rather than the original collection locale; “gbif” and “institutions” tests). Records with the same latitude and longitude or zero values were also excluded.

**Figure S1:** Next page. For phylogenetically informed Bayesian multi-level models, implemented using the brms package (v. 2.15.0; Bürkner, 2017), weakly informative priors were constructed using modified versions of default priors and subsequently vetted using prior predictive checks. Priors for each model, formatted in brms output, are shown.

**Results S1:**

Petasoid echinate

*Code:* PET_ECH

*Rationale for naming:* Because the taxonomic occurrence and anatomical origin of this type cannot always be certain, it is given a morphological name. The term ‘petasoid’ designates a hat-shaped body, from the Latin word for hat (“petasus”) and the term “echinate” refers to the small echina covering the central part of the “hat.”

*Description:* Small, flat disc with the central part elevated into a hemi-spheroidal (rounded) bump or mound. Disc typically circular in planar view with entire, granulate, or irregular edge; in side view, disc resembles a bowler hat or flying saucer with a flat underside and a convex center. On the surface of the central mound are several (~3 or more) small conical projections, typically with rounded apices. Surface texture is psilate to granulate. Occurring singly or in articulated rows.

*Size:* Generally ~3-25 µm in diameter (Tomlinson, 1961; Runge, 1999; this study).

*Anatomical origin and taxonomic occurrence:* In palms and orchids, specialized silica-producing parenchyma cells, so called stegmata of German authors (e.g., Molish, 1913; Tomlinson, 1961). Stegmata are found in longitudinal continuous or discontinuous files of cells adjacent to fibers sheathing vascular bundles, phloem of vascular bundles, or to non-vascular sclerenchyma fibers (Tomlinson, 1961; Prychid et al., 2004). In Marantaceae, they can also be found in the leaf mesophyll underlying the palisade parenchyma (Prychid et al., 2004).

Petasoid echinate is found in abundance in the leaf, stem, root, and reproductive structures of palms in the Nypoideae, certain tribes in Ceroxyloideae (Hypophorbeae), Arecoideae (Caryoteae, Triarteae, two subtribes within Areceae, one subtribe within Cocoeae) (e.g., Tomlinson, 1961; Kondo and Sase, 1986; Piperno, 1985, 1988; Strömberg, 2003; Prychid et al., 2004; this study). It has also been reported from the tissues of Orchidaceae and Marantaceae (vegetative tissues only) (Piperno, 1985, 1988; Kondo and Sase, 1986; Prychid et al., 2004; Chen and Smith, 2013; this study).

*Discussion and interpretation:* Petasoid echinate is found abundantly in certain palms and is therefore most commonly used to indicate palms, such as the mangrove palm, *Nypa* (e.g., Das et al., 2013; Chabot et al., 2018).

Hat-shaped phytoliths in Lowiaceae (Chen and Smith, 2013) fall outside of the morphological range defined for Petasoid echinate herein because of their dentate edge and tall protrusion and long, pointy projections. Petasoid echinate differ from the epidermal plate phytoliths of Cyperaceae, which tends to have one main, well-defined, conical or hemi-spheroidal structure (the surface of which can be papillar) in the center of the plate; in addition, the sedge plate can be polygonal or rectangular shape rather than circular.

*Synonyms:* Stegmata or cover cells (“Deckzellen”) (Molish, 1913: p. 73-74); Hat-shaped bodies (Tomlinson, 1961: Plate 8B). Hat-shaped spinulose phytoliths (Piperno 1985: Plate II, Fig. 1, V, Fig. 4, VI, Fig. 1). Conical to hat-shaped bodies (Piperno, 1988: Plate 21, 38-40). Rugulose, conical phytoliths (Kealhofer & Piperno, 1998: Fig. 12). Conical or hat-shaped stegmata phytolith (D5) (Runge, 1999). *Nypa*-type body: bowler hat with tubercles (Clm-1) (Strömberg, 2003: Fig. 4.10j). Conical silica bodies with truncated tops (hat shaped) (Prychid et al., 2004: Fig. 2A).

*Illustrations:* A. *Guarianthe aurantiacasee*, Orchidaceae (UWBM PR 7859), B. *Cephalanthera rubra*, Orchidaceae (UWBM PR 7149), C, D. *Sclerosperma walkeri*, Arecaceae-Arecoideae-Sclerospermeae (UWBM PR 41499), E, F. *Nypa fruticans*, Arecaceae-Nypaoideae (UWBM PR 6010; UCMP 399328), G, H. *Socratea exorrhiza*, Arecaceae-Arecoideae-Iriarteeae (UWBM PR 7002), I, J. *Caryota mitis*, Arecaceae-Coryphoideae-Caryoteae (UWBM PR 6639), K. Fossil from TUR2, Middle Miocene of Turkey (UWBM PB 13499). See also main text Figs. 1a & 2.

**Figure S2:** Phylomorphospace showing species average phytolith morphology for the entire palm family. In the lefthand panel four taxonomic groups are highlighted, and in the righthand panel intraspecific variation observed in four species from those taxonomic groups is shown. Species were semi-arbitrarily chosen to aid in visualisation, but broadly reflect patterns observed in the broader dataset (i.e., intraspecific variation in selected species was not notably higher than the remaining species). Axes show the first and second principal component axes obtained via principal component analysis on quantitative phytolith measures used in discriminant analyses. Respectively these explained 48% and 28% of the variation in these traits.

**Figure S3:** Summary of discriminant analysis results for prediction of Spheroid echinate phytoliths only. The panel on the left shows variation in overall accuracy for a range of methods and data treatments, while the panel on the right shows Cohen’s κ for the same models. Results show that models trained only on spheroid echinate phytoliths do not substantially outperform those trained using the full dataset (i.e., including both hat-shaped and intermediate phytoliths) in either metric. Notably, the “standard RF model” (red triangles) is the best performing in both metrics (see main text). Note that the full training set models for each method (both red and green points) are the same as those shown in Fig. 3a of the main text.

**Figure S4:** Summary of discriminant analysis results for prediction of hat-shaped phytoliths only. The panel on the left shows variation in overall accuracy for a range of methods and data treatments, while the panel on the right shows Cohen’s κ for the same models. Results show that Random Forest (RF) models trained only on hat-shaped phytoliths do not substantially outperform those trained using the full dataset (i.e., including both Spheroid echinate and intermediate phytoliths). This contrasts with several other methods, notably LDA and SVM, where limiting the training set to just hat-shaped phytoliths does substantially improve results. The “standard RF model” (in red, see main text) is the joint best performing in both metrics, with the same number of phytoliths correctly identified as hat-only Random Forest models and comparable Cohen’s κ. Note that the full training set models for each method (both red and green points) are the same as those shown in Fig. 3a of the main text.

**Figure S5:** Comparison of results from standard models (i.e., all morphotypes combined) with an ensemble of the best models trained separately for hat-shaped (Fig. S4) and Spheroid echinate (Fig. S3) phytoliths. Both models used to create the ensemble were trained using a Random Forest. The figure shows results for individual taxonomic groups, indicating number of phytoliths, number of model guesses, and number of phytoliths correctly identified for the “standard RF model”, and the ensemble model. Both treatments returned similar results both overall and considering each individual taxonomic group. In total, the ensemble model correctly identified 443 of 753 phytoliths (58.8 %) in the test set, while the standard RF model correctly identified 445 (59.1 %).

**Figure S6:** Ancestral state estimations of mean annual precipitation (MAT, °C), precipitation of the dryest quarter (DQP, cm), and evergreen forest affinity (%) for taxa for which we obtained both phytolith and climate data. Ancestral state estimates were based upon a dataset compiled for 1017 species, and pruned to show only those represented in the phytolith dataset.

**Figure S7:** Posterior estimates with 95% credibility intervals from phylogenetically informed multilevel models, showing the effect (i.e., slope) of all five environmental predictors (MAT, QMT, MAP, DQP, EFA) on the seven quantitative phytolith measurements included in our analyses. Environmental predictor acronyms are as follows: MAT (mean annual temperature, °C), CMT (cold month temperature, °C), MAP (mean annual precipitation, cm), DQP (dry quarter precipitation, cm), and EFA (evergreen forest affinity, %). Estimates with 95% credibility intervals overlapping zero (i.e., no effect of predictor) are shown in black, while those excluding zero are shown in red. The latter included only the weak effects of CMT on variation in tubercle height and DQP on phytolith circularity (see main text).

**Figure S8:** Discriminant model results for Random Forest models predicting phytolith subfamily (for all palms and family for outgroup taxa). The lefthand panel shows overall model accuracy, with the red-shaded region indicating values less than or equal to the no information rate. The righthand panel shows the confusion matrix for the same model, with degree of shading representing the relative number of phytoliths per group assigned to each group, where columns represent model guesses, and rows represent true phytolith identity. Shading off the diagonal therefore represents incorrect model assignments.

**Figure S9:** Discriminant model results for Random Forest models predicting phytolith tribe (for all palms and family for outgroup taxa). The lefthand panel shows overall model accuracy, with the red-shaded region indicating values less than or equal to the no information rate. The righthand panel shows the confusion matrix for the same model, with degree of shading representing the relative number of phytoliths per group assigned to each group, where columns represent model guesses, and rows represent true phytolith identity. Shading off the diagonal therefore represents incorrect model assignments.

REFERENCES:

Chabot, Y., Garnier, A., Geria, I.M., 2018. Phytolith analysis from the archaeological site of Kota Cina (North Sumatra, Indonesia). Journal of Archaeological Science: Reports 20, 483-501.

Chen, S.T., Smith, S.Y., 2013. Phytolith variability in Zingiberales: A tool for the reconstruction of past tropical vegetation. Palaeogeography Palaeoclimatology Palaeoecology 370, 1-12.

Das, S., Ghosh, R., Bera, S., 2013. Application of non-grass phytoliths in reconstructing deltaic environments: A study from the Indian Sunderbans. Palaeogeography Palaeoclimatology Palaeoecology 376, 48-65.

Kealhofer, L., Piperno, D., 1998. Opal phytoliths in Southeast Asian flora. Smithsonian Contributions to Botany 88, 1-39.

Kondo, R., Sase, T., 1986. Opal phytoliths, their nature and application. The Quaternary Research 25, 31-63.

Molisch, H., 1913. Michrochemie der Pflanzen. Jena.

Piperno, D.R., 1985. Phytolith analysis and tropical paleo-ecology: Production and taxonomic significance of siliceous forms in New World plant domesticates and wild species. Review of Palaeobotany and Palynology 45, 185-228.

Piperno, D.R., 1988. Phytolith Analysis, an Archaeological and Geological Perspective. Academic Press, San Diego.

Prychid, C.J., Rudall, P.J., Gregory, M., 2004. Systematics and biology of silica bodies in monocotyledons. The Botanical Review 69, 377-440.

Runge, F., 1999. The opal phytolith inventory of soils in central Africa - quantities, shapes, classification, and spectra. Review of Palaeobotany and Palynology 107, 23-53.

Tomlinson, P.B., 1961. Anatomy of the Monocotyledons, Volume II, Palmae. Oxford University Press, London
